# The discrete ordinate method for angular flux calculations in slab geometry

**DOI:** 10.1016/j.heliyon.2019.e02211

**Published:** 2019-08-05

**Authors:** M. Lahdour, T. El Bardouni, M. Mohammed, S. El Ouahdani

**Affiliations:** a Radiations and Nuclear Systems Laboratory, University Abdelmalek Essaadi, Faculty of Sciences of Tetuan, Morocco; b ERPTM laboratory, Department of Physics, Sultan Moulay Slimane University, Beni Mellal, Morocco

**Keywords:** Nuclear physics, Neutron transport equation, MCNP6, Angular flux

## Abstract

In many deterministic methods, concerning the resolution of the neutron transport equation, a more global and practical representation of the angular flux is needed to provide us with useful and complete information on the neutron population in a reactor core. The purpose of this paper is to provide a reference quality calculation for the angular flux. The discrete ordinate method (S${}_{\text{N}}$) is processed with a new matrix form which is used to model an isotropic and anisotropic multiplicative system in a region, multi-region for one energy group in a cartesian geometry. The obtained results are compared to those obtained by MCNP6 code.

## 1 Introduction

The neutron transport equation describes the neutron population in a reactor (Stamm'ler and Abbate, 1983). The prediction of a correct neutron population is very important to have a safer and more economical reactor design. For purposes of steady state reactor core design, we seek a solution for the one speed time-independent linear neutron transport equation. The equation is essentially a statement of neutron balance with the angular neutron flux $\psi (\overset{\to }{r},\overset{\to }{\Omega })$ as the principle unknown, i.e.

(1) $$\overset{\to }{\Omega }\cdot \overset{\to }{\nabla }\psi (\overset{\to }{r},\overset{\to }{\Omega })+\Sigma (\overset{\to }{r})\psi (\overset{\to }{r},\overset{\to }{\Omega })=Q(\overset{\to }{r},\overset{\to }{\Omega })$$

Where $\psi (\overset{\to }{r},\overset{\to }{\Omega })$ is the angular flux of particles, $\Sigma (\overset{\to }{r})$ is the macroscopic total cross section and $Q(\overset{\to }{r},\overset{\to }{\Omega })$ is the particle source distribution. The angular flux is defined as a product of the neutron speed *v* and the neutron angular density (Lewis and Miller, 1984). The idea of the discrete ordinates approach is to approximate the angular flux at a set of spatial mesh points and in a number of fixed directions. The directions are often denoted as ordinates giving the discretization scheme its names. Also the discrete ordinates method widely considered as the dominant means for obtain numerical solutions to the integro-differential form of the transport equation has been used extensively in reactor core calculations. In this method, after selecting a discretization for the angular variable in a number *N* of Gauss-Legendre quadrature points, we perform a discretization for the *r* spatial variable. The most used technique consists of replacing the differential terms in Eq. (2), with finite difference relations and the angular flux in the collision terms by the diamond difference (DD) relations; then the source term including the scattering and fission source will be expressed in the Legendre polynomial base.

Our general plan is to describe the discrete ordinates approach in more details with matricial formulation in Section 2, and certain numerical comparisons of our S${}_{\text{N}}$ method with MCNP6 code (Goorley et al., 2012) in Section 3. We summarize and discuss extensions of the work in Section 4.

## 2 Materials & methods

### 2.1 The S${}_{\text{N}}$ method

The first application of the discrete ordinates method was in the astrophysics field by Chandrasekhar (1960), after it was used in the radiative transfer studies by Lathrop (1966). The S${}_{\text{N}}$ method is a special case of the discrete ordinates method. It was firstly introduced by Carlson (1955) in the context of reactor physics for the computation of particle transport. The S${}_{\text{N}}$ method or discrete ordinates is a collocative method. It uses the integro-differential form of the transport equation and involves a special treatment of the angular variable. The treatment of spatial variables is often based on a finite difference scheme called a diamond scheme. This is why it is relatively common in neutronics that the term S${}_{\text{N}}$ method implicitly implies a diamond difference scheme. For the sake of brevity, we limit our discussion to steady-state transport equation for monoenergetic neutrons in a slab geometry (Lahdour et al., 2019) for which the transport equation is reduced to

(2) $$\mu \frac{d\psi (x,\mu )}{dx}+\Sigma_{t}(x)\psi (x,\mu )=Q(x,\mu )$$

where:

ψ(x,μ): the angular flux of the neutrons at position *x* traveling in direction *μ*.

*μ*: cosine of the angle between the neutron velocity vector and the positive x-axis.

$\Sigma_{t}(x)$: the total macroscopic cross-section.

Q(x,μ): the source density which is only dependent upon *x* and *μ*.

The source density in one-energy group is related to the Legendre coefficients of the flux by:

(3) $$Q(x,\mu )=\sum_{l=0}^{L}\frac{2l+1}{2}\left[\Sigma_{s,l}(x)P_{l}(\mu )\phi_{l}(x)+\frac{1}{k_{eff}}\chi \nu \Sigma_{f}(x)\phi_{l}(x)\delta_{l0}\right]$$

where:

$\phi_{l}(x)$: the Legendre coefficients of the flux.

$\Sigma_{s,l}(x)$: the Legendre coefficients of the scattering macroscopic cross-section (or Legendre moments).

*L*: large integer number.

$P_{l}(\mu )$: the Legendre polynomials.

However, for this study, *L* will be either 0, 1 or 2. L=0 for $P_{0}$ scattering, L=1 for $P_{1}$ scattering and L=2 for scattering $P_{2}$.

For simplification, we adopt that the fission neutron angular distribution is isotropic in laboratory coordinates and further, the fission cross section $\Sigma_{f}$ is independent of the initial neutron direction, *ν* is an average neutrons number emitted by fission and *χ* is the fission spectrum. The Kronecker delta $\delta_{l0}$ is introduced in the fission term to take into account the isotropy of fission source. In order to solve the Eq. (2), we define *N* discrete directions ($\mu_{1},\mu_{2},...,\mu_{N}$) and corresponding weight coefficients ($w_{1},w_{2},...,w_{N}$). These weights are chosen to approximate the integral of any function f(μ) by a weighted sum:

(4) $$\int_{-1}^{1}f(\mu )d\mu \approx \sum_{n=1}^{N}f(\mu_{n})w_{n},$$

(5) $$\sum_{n=1}^{N}w_{n}=2.$$

**Figure 1:**
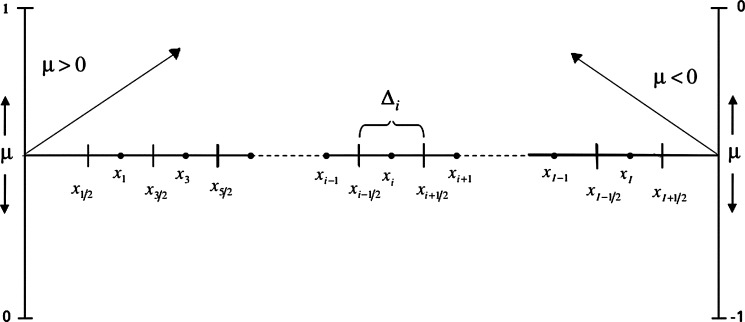
Spatial discretization in discrete ordinates method.

Then we consider a Slab geometry with a number of mesh *I* and outer vacuum boundary conditions (see Fig. 1). Using this discretization of the angular coordinate and space, integrating Eq. (2) over each cell $\Delta_{i}$, the Eq. (2) will be written as:

(6) $$\mu_{n}(\psi_{i+1/2,n}-\psi_{i-1/2,n})+\Delta_{i}\Sigma_{t,i}\psi_{i,n}=\Delta_{i}Q_{i,n}$$

In the choice of *μ*, standard approaches based on Gaussian quadrature rules are implemented for the two intervals [−1,0] and [0,1] or *N* Gauss-Legendre points are selected on [−1,1]. *N* is an even number chosen for symmetry purpose with respect to μ=0, this choice is due to the intent to assign the same importance particles which streaming along different directions. For any direction $\mu_{n}$, the Eq. (6) represents a system of *I* equations with three unknowns $\psi_{i-1/2,n}$, $\psi_{i,n}$ and $\psi_{i+1/2,n}$ in each cell of width $\Delta_{i}$ for neutrons traveling in angular cosine $\mu_{n}$ (see Fig. 1). The angular cosine $\mu_{n}$ sign indicates the endpoint flux direction either “incoming” or “outgoing” particles. With a process called “sweeping” (Lewis and Miller, 1984), the incoming fluxes can be considered as known quantities. So the unknowns are $\psi_{i,n}$ and the outgoing flux. Hence, for segment $\Delta_{i}$, we have two unknowns, so two equations are required. The neutron balance Eq. (6) provides one equation. In the aim to eliminate the residual unknown the so-called diamond rule is used.

(7) $$\psi_{i,n}=\frac{1}{2}(\psi_{i+1/2,n}+\psi_{i-1/2,n})$$

The decoupled hyperbolic character of Eq. (2) allows straightforward determination of inflow and outflow boundaries. Boundary conditions (Stamm'ler and Abbate, 1983) are then easily taken into account i.e., they can be zero incoming flux at the surface of the medium. In order to apply the strategy described above, the discrete directions are rearranged so that, for n=1 to N/2, with $\psi_{i+1/2,n}=2\psi_{i,n}-\psi_{i-1/2,n}$ (sweeping to the right), $\mu_{n}$ corresponds to a set of positive directions and for n=N/2+1 to *N* with $\psi_{i-1/2,n}=2\psi_{i,n}-\psi_{i+1/2,n}$ (sweeping to the left), $\mu_{n}$ is relative to the negative directions. Consequently, Eq. (6) is subdivided into the following two equations:

(8) $$\left\{ \begin{array}{cc} A_{i,n}^{+}\psi_{i,n}+B_{i,n}^{+}\psi_{i-1/2,n}=Q_{i,n} & \text{for }n=1,...,N/2,\\ A_{i,n}^{-}\psi_{i,n}+B_{i,n}^{-}\psi_{i+1/2,n}=Q_{i,n} & \text{for }n=N/2+1,...,N \end{array}\right.$$

where

(9) $$A_{i,n}^{\pm }=\frac{\Sigma_{t,i}\Delta_{i}\pm 2\mu_{n}}{\Delta_{i}},$$

and

(10) $$B_{i,n}^{\pm }=\mp 2\frac{\mu_{n}}{\Delta_{i}}$$

These equations can be written for S${}_{\text{2}}$ method and *I* meshes in the following matrix form:

(11) $$\begin{array}{c} \left(\begin{array}{cccccccccc} A_{1,1}^{+}\; & 0\; & 0\; & 0\; & 0\; & 0\; & 0\; & 0\; & 0\; & 0\\ 2B_{2,1}^{+}\; & A_{2,1}^{+}\; & 0\; & 0\; & 0\; & 0\; & 0\; & 0\; & 0\; & 0\\ -2B_{3,1}^{+}\; & 2B_{3,1}^{+}\; & A_{3,1}^{+}\; & 0\; & 0\; & 0\; & 0\; & 0\; & 0\; & 0\\ \ddots \; & \ddots \; & \ddots \; & \ddots \; & 0\; & 0\; & 0\; & 0\; & 0\; & 0\\ -2B_{I,1}^{+}\; & 2B_{I,1}^{+}\; & -2B_{I,1}\; & 2B_{I,1}^{+}\; & A_{I,1}^{+}\; & 0\; & 0\; & 0\; & 0\; & 0\\ 0\; & 0\; & 0\; & 0\; & 0\; & A_{I,2}^{-}\; & 0\; & 0\; & 0\; & 0\\ 0\; & 0\; & 0\; & 0\; & 0\; & 2B_{I-1,2}^{-}\; & A_{I-1,2}^{-}\; & 0\; & 0\; & 0\\ 0\; & 0\; & 0\; & 0\; & 0\; & -2B_{I-2,2}^{-}\; & 2B_{I-2,2}^{-}\; & A_{I-2,2}^{-}\; & 0\; & 0\\ 0\; & 0\; & 0\; & 0\; & 0\; & \ddots \; & \ddots \; & \ddots \; & \ddots \; & 0\\ 0\; & 0\; & 0\; & 0\; & 0\; & -2B_{1,2}^{-}\; & 2B_{1,2}^{-}\; & -2B_{1,2}^{-}\; & 2B_{1,2}^{-}\; & A_{1,2}^{-} \end{array}\right) \end{array}\begin{array}{c} \left(\begin{array}{c} \psi_{1,1}\\ \psi_{2,1}\\ \psi_{3,1}\\ \vdots \\ \psi_{I,1}\\ \psi_{I,2}\\ \psi_{I-1,2}\\ \psi_{I-2,2}\\ \vdots \\ \psi_{1,2} \end{array}\right) \end{array}\;\;=\begin{array}{c} \left(\begin{array}{c} Q_{1,1}\\ Q_{2,1}\\ Q_{3,1}\\ \vdots \\ Q_{I,1}\\ Q_{I,2}\\ Q_{I-1,2}\\ Q_{I-2,2}\\ \vdots \\ Q_{1,2} \end{array}\right) \end{array}$$

So a general matrix form of the S${}_{\text{N}}$ method in a slab geometry is given by:

(12) Aψ=Q,

where *ψ* and *Q* are the angular flux vector and emission density vector respectively and *A* is a diagonal matrix. Eq. (12) can be written as:

(13) $$\begin{array}{c} \left(\begin{array}{cccccccccc} M_{1}^{+} & 0 & 0 & 0 & 0 & 0 & 0 & 0 & 0 & 0\\ 0 & M_{2}^{+} & 0 & 0 & 0 & 0 & 0 & 0 & 0 & 0\\ 0 & 0 & M_{3}^{+} & 0 & 0 & 0 & 0 & 0 & 0 & 0\\ 0 & 0 & 0 & \ddots & 0 & 0 & 0 & 0 & 0 & 0\\ 0 & 0 & 0 & & M_{N/2}^{+} & 0 & 0 & 0 & 0 & 0\\ 0 & 0 & 0 & 0 & 0 & M_{N/2+1}^{-} & 0 & 0 & 0 & 0\\ 0 & 0 & 0 & 0 & 0 & 0 & M_{N/2+2}^{-} & 0 & 0 & 0\\ 0 & 0 & 0 & 0 & 0 & 0 & 0 & M_{N/2+3}^{-} & 0 & 0\\ 0 & 0 & 0 & 0 & 0 & 0 & 0 & 0 & \ddots & 0\\ 0 & 0 & 0 & 0 & 0 & 0 & 0 & 0 & 0 & M_{N}^{-} \end{array}\right)\left(\begin{array}{c} \psi_{1}^{+}\\ \psi_{2}^{+}\\ \psi_{3}^{+}\\ \vdots \\ \psi_{N/2}^{+}\\ \psi_{N/2+1}^{-}\\ \psi_{N/2+2}^{-}\\ \psi_{N/2+3}^{-}\\ \vdots \\ \psi_{N}^{-} \end{array}\right) \end{array}\;\;=\begin{array}{c} \left(\begin{array}{c} Q_{1}^{+}\\ Q_{2}^{+}\\ Q_{3}^{+}\\ \vdots \\ Q_{N/2}^{+}\\ Q_{N/2+1}^{-}\\ Q_{N/2+2}^{-}\\ Q_{N/2+3}^{-}\\ \vdots \\ Q_{N}^{-} \end{array}\right) \end{array}$$

Each element of matrix *A* contains a set of components $M_{n}^{+}$ or $M_{n}^{-}$ according to the positive or negative neutron direction respectively. Also each element of emission density vector *Q* contains a set of components $Q_{n}^{+}$ or $Q_{n}^{-}$ according to the positive or negative neutron direction respectively. The matrices $M_{n}^{+}$ and $M_{n}^{-}$ are lower triangular type.

(14) $$M_{n}^{+}=\left(\begin{array}{ccccc} A_{1,n}^{+} & 0 & 0 & 0 & 0\\ 2B_{2,n}^{+} & A_{2,n}^{+} & 0 & 0 & 0\\ -2B_{3,n}^{+} & 2B_{3,n}^{+} & A_{3,n}^{+} & 0 & 0\\ \ddots & \ddots & \ddots & \ddots & 0\\ -2B_{I,n}^{+} & 2B_{I,n}^{+} & -2B_{I,n} & 2B_{I,n}^{+} & {A_{I,n}}^{+} \end{array}\right),\psi_{n}^{+}=\left(\begin{array}{c} \psi_{1,n}\\ \psi_{2,n}\\ \psi_{3,n}\\ \vdots \\ \psi_{I,n} \end{array}\right);\;n=1,...,N/2$$

(15) $$M_{n}^{-}=\left(\begin{array}{ccccc} A_{I,2}^{-} & 0 & 0 & 0 & 0\\ 2B_{I-1,2}^{-} & A_{I-1,2}^{-} & 0 & 0 & 0\\ -2B_{I-2,2}^{-} & 2B_{I-2,2}^{-} & A_{I-2,2}^{-} & 0 & 0\\ \ddots & \ddots & \ddots & \ddots & 0\\ -2B_{1,2}^{-} & 2B_{1,2}^{-} & -2B_{1,2}^{-} & 2B_{1,2}^{-} & {A_{1,2}}^{-} \end{array}\right),\psi_{n}^{-}=\left(\begin{array}{c} \psi_{I,n}\\ \psi_{I-1,n}\\ \psi_{I-2,n}\\ \vdots \\ \psi_{1,n} \end{array}\right);\;n=N/2+1,...,N$$

Eq. (16) gives the relationship between the angular flux and the scalar flux.

(16) $$\phi_{i}=\sum_{n=1}^{N}w_{n}\psi_{i,n}$$

**Figure 2:**
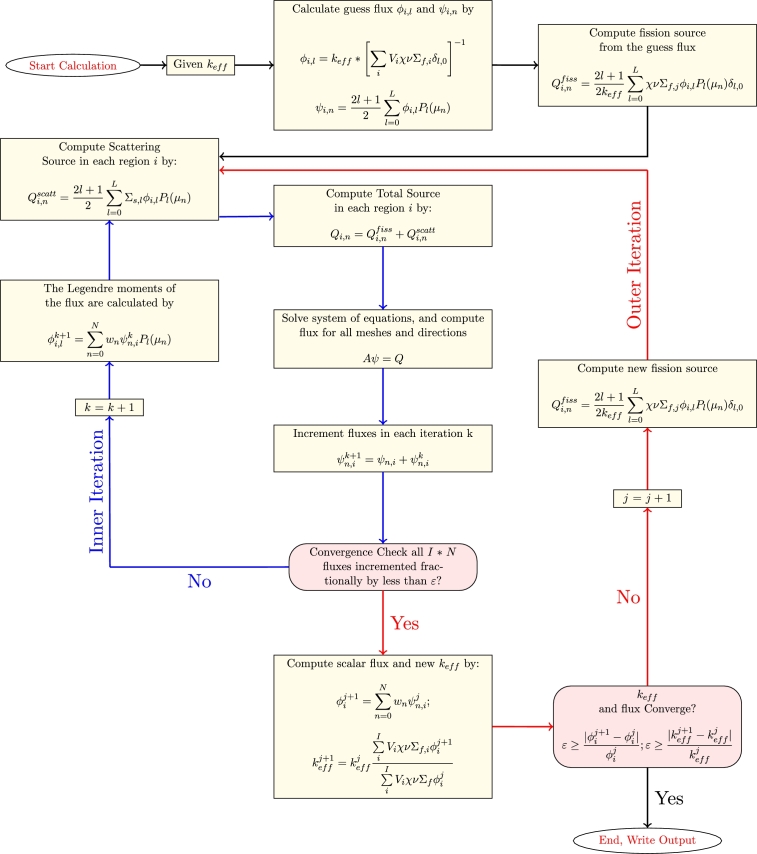
S${}_{\text{2}}$ matrix sparsity pattern for a 10 × 10 × 2 spatial mesh and one energy group.

**Figure 3:**
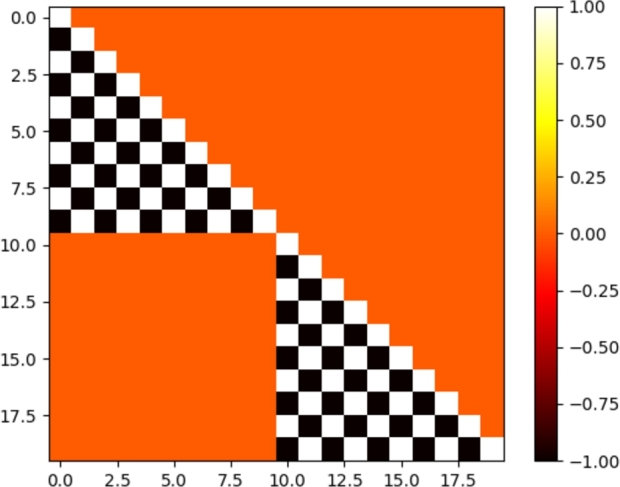
S${}_{\text{8}}$ matrix sparsity pattern for a 10 × 10 × 8 spatial mesh and one energy group.

The S${}_{\text{N}}$ approximation for one-speed neutrons in slab geometry generates equations system which is casted in triangular matrix per block. Each block corresponds to a direction of dimension I×I. The matrix system form's (12) does not require the inversion of the matrix *A*, it has been solved by an iterative process called forward substitution for lower triangular matrices. This system has been divided into two sub triangular matrix $M_{n}^{+}$ and $M_{n}^{-}$ for a computer income. In our calculation, we considered a slab geometry with the vacuum at the boundary, which allowed us to carry out a transport “sweep” only for the positive or negative directions of the neutrons, consequently the sub-triangular matrices $M_{n}^{+}$ and $M_{n}^{-}$ are symmetrical, that is, we can calculate $M_{n}^{-}$ using Eq. (9) and (10) and then deduce $M_{n}^{+}$ from $M_{n}^{-}$ and vice versa, which makes the calculation faster. The sparsity plot of a representative *A* matrix for a 10×10×2 and 10×10×8 grid with vacuum boundary conditions is shown in Figs. 2 and 3 respectively.

**Figure 4:**
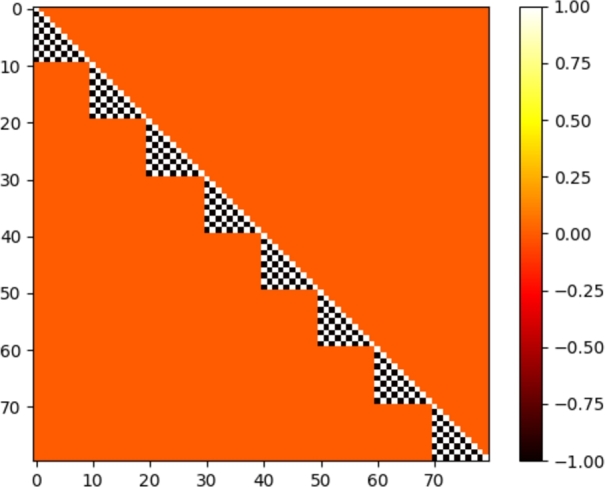
Diagram of criticality calculation by *S*_*N*_ method.

Fig. 4 summarizes a diagram of S${}_{\text{N}}$ calculation method, into a slab geometry, which is the base of our new developed deterministic code. The effective neutron multiplication factor k${}_{eff}$ and the flux distribution for a medium (single region or multi-regions) are obtained using the conventional inner-outer iteration method. Before starting the iterations, the guess value of moments and angular fluxes are estimated in all meshes for a given value of multiplication factor (k${}_{eff}$). At the beginning of an external iteration, the fission source and scattering source are calculated in all meshes and directions from the guess moments flux. Starting from scattering source in inner iteration, the total source as defined in Eq. (6) is calculated in all meshes and directions. Then, for each internal iteration, the matrix system defined in the Eq. (12) is solved by an iterative process called direct substitution for a lower triangular matrix. The angular flux is converged by checking that all (I×N) fluxes incremented fractionally by less than $10^{-8}$ in each inner iteration. Also, in the inner iteration, we are interested in updating the scattering source whereas the external iterations were used to update the fission source until reaching the convergence on the multiplication factor and the scalar flux is calculated by collecting all the average angular fluxes.

## 3 Results & discussion

### 3.1 One region criticality results

In this section, the developed S${}_{\text{N}}$ method was verified on four Benchmark cases and validated by comparing our results to those calculated by MCNP6 code (Goorley et al., 2012). The two first Benchmark cases, refer to a single region slab, and one energy group with isotropic scattering source problem. The last two cases, consist of a single region slab, one-speed particles with scattering source anisotropy. These Benchmarks are chosen to verify the S${}_{\text{N}}$ algorithm with different behavior of the scattering source; and they are motivating for the fact that we can calculate easily the angular flux. The corresponding macroscopic cross sections used in both MCNP6 and S${}_{\text{N}}$ calculations are taken from Sood et al. (2003) and illustrated in Table 1, Table 2, Table 3. The input parameters that we have adopted to perform our calculation are defined as follows: in the S${}_{\text{N}}$ method, the convergence criterion on the scalar flux and the multiplication factor is $10^{-8}$, the fine mesh number in each region is 1000 with the angular quadrature N=200. For Monte Carlo calculation, the first 150 batches were skipped and followed by 5000 active batches with 1000000 particles per batch. The estimated statistical errors were reduced to 3 pcm for effective multiplication factor (k${}_{eff}$) values and less than 0.2% for current calculations. For MCNP6 simulations, the space and angular variables were discretized in order to calculate the angular neutron current at each interface on 101 space points and 202 directions from left to right. Comparison has been made for eight selected MCNP6 angular flux curves matching eight directions of the Gauss-Legendre quadrature formula used in S${}_{\text{N}}$ calculation. For normalization purpose formula (17) was used, with <*μ*> is the direction of neutron, $\overset{\to }{j_{-}}(x)$ is the neutron current in position *x* and Δ*μ* is the interval width between two succeeding directions where MCNP6 angular neutron current is calculated.

(17) $$\psi (x,<\mu >)≃\frac{\overset{\to }{j_{-}}(x)}{<\mu >\Delta \mu }$$

where

(18) $$<\mu >=\frac{\left|\mu_{i}-\mu_{i+1}\right|}{2},\;\;i=1,...,8$$

*i* is the neutron direction index matching those of the quadrature formula.

**Table 1:** Description of criticality verification suite problems.

Problem	Benchmark identifier	r_*c*_ (cm) critical dimension	Description
2	PUa-1-0-SL	1.853722	semi-infinite slab with P_0_ scattering cross sections
22	UD2O-1-0-SL	10.371065	semi-infinite slab with P_0_ scattering cross sections
32	PUa-1-1-SL	0.77032	semi-infinite slab with P_1_ scattering cross sections
33	PUa-1-2-SL	0.76378	semi-infinite slab with P_2_ scattering cross sections

**Table 2:** Anisotropic cross section data.

Material	*ν*	Σ_*f*_	Σ_*c*_	Σ_*s*0_	Σ_*s*1_	Σ_*s*2_	Σ_*t*_
Pu-239 (a)	2.5	0.266667	0.0	0.733333	0.20	0.075	1.0

**Table 3:** Isotropic cross section data.

Material	*ν*	Σ_*f*_	Σ_*c*_	Σ_*s*_	Σ_*t*_
Pu-239 (a)	3.24	0.081600	0.019584	0.225216	0.32640
U-D_2_O	1.70	0.054628	0.027314	0.464338	0.54628

**Figure 5:**
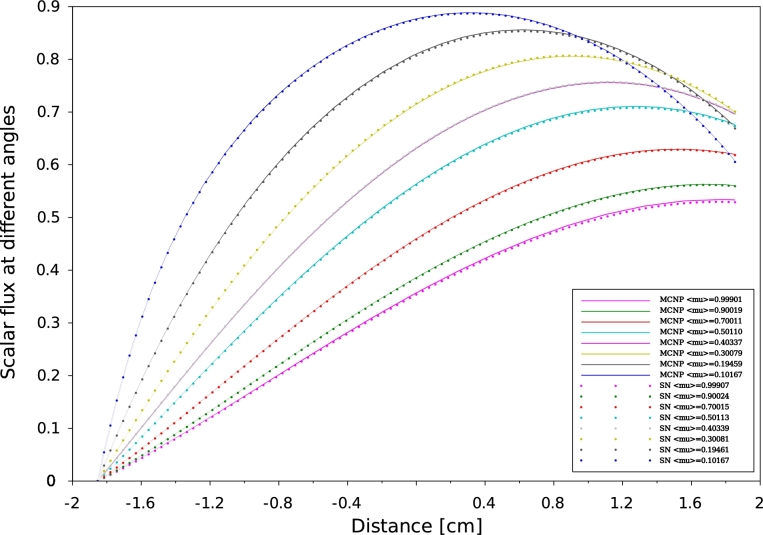
Scalar flux distribution at different angles for problem 2 MCNP6 vs S${}_{\text{N}}$ for neutrons traveling from left to right.

**Figure 6:**
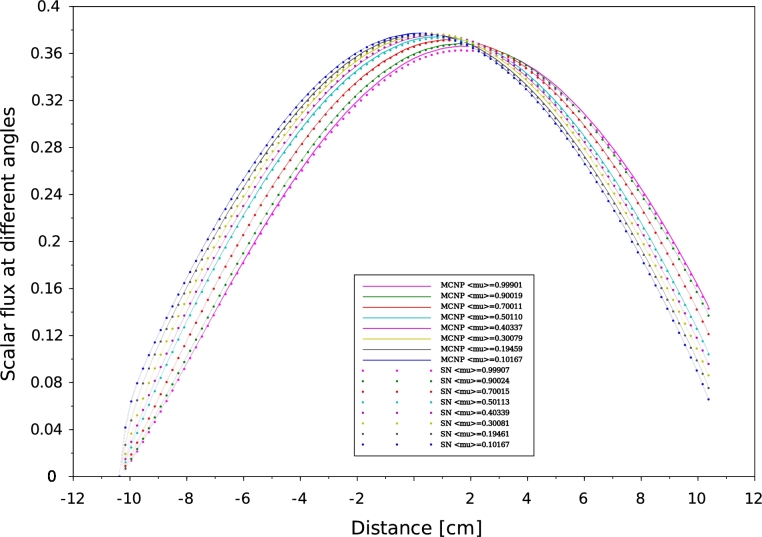
Scalar flux distribution at different angles for problem 22 MCNP6 vs S${}_{\text{N}}$ for neutrons traveling from left to right.

**Figure 7:**
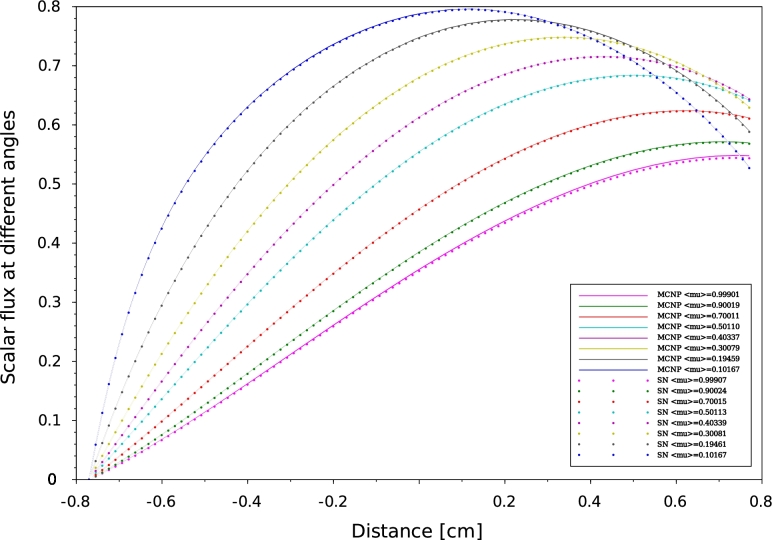
Scalar flux distribution at different angles for problem 32 MCNP6 vs S${}_{\text{N}}$ for neutrons traveling from left to right.

**Figure 8:**
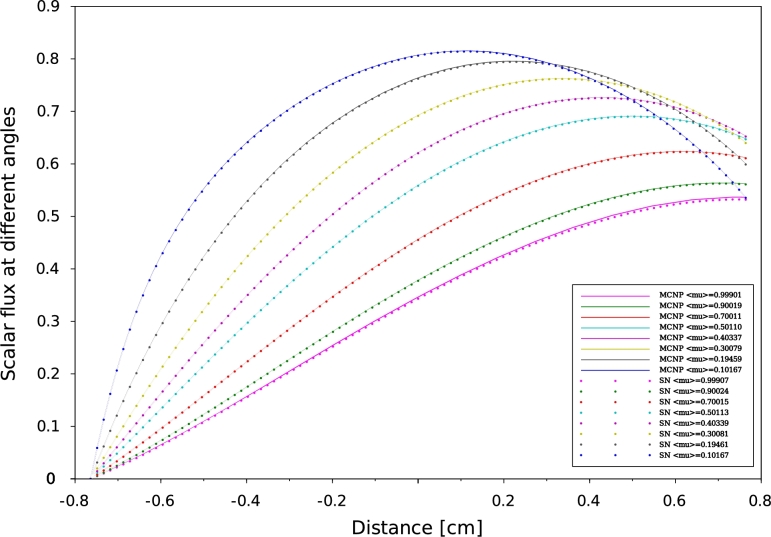
Scalar flux distribution at different angles for problem 33 MCNP6 vs S${}_{\text{N}}$ for neutrons traveling from left to right.

The effective multiplication factor k${}_{eff}$ obtained with the S${}_{\text{N}}$ method and those of the references namely, Analytical calculation (Sood et al., 2003) and MCNP6 code (Goorley et al., 2012) are summarized in Table 4. The S${}_{\text{N}}$ scalar flux at different angles results are compared with that calculated by MCNP6 code and shown in Figs. 5, 6, 7 and 8. The simulations are run on my personal computer (Intel Core i5 (2nd Gen) 2520M/2.5 GHz) and the required average time for each simulation was a 120 seconds.

**Table 4:** k${}_{eff}$ multiplication factor of the investigated criticality verification suite problems.

Case	Analytic	S${}_{\text{N}}$		MCNP6	
	k${}_{eff}$	k${}_{eff}$	Relative error %	k${}_{eff}$	Relative error %
PUa-1-0-SL	1.00000	0.99999	±10 ⋅ 10^−4^	1.00000	±0.0
UD2O-1-0-SL	1.00000	1.00000	±0.0	1.00002	±20 ⋅ 10^−4^
PUa-1-1-SL	1.00000	1.00001	±10 ⋅ 10^−4^	1.00001	±10 ⋅ 10^−4^
PUa-1-2-SL	1.00000	0.99998	±20 ⋅ 10^−4^	1.00001	±10 ⋅ 10^−4^

### 3.2 Multi-region criticality results

**Figure 9:**
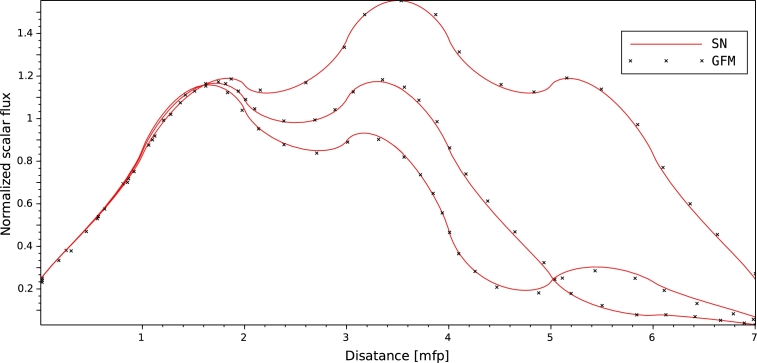
Normalized scalar fluxes in multi-region slab geometry.

The second test problem is a heterogeneous, one-group, seven-region slab with outer surface vacuum boundary conditions and isotropic scattering source. The test aims to determine the effective multiplication factor and scalar flux of the slab. The multi-region geometry consists of seven regions arranged in three different configurations of fuel, reflector and absorber (Table 5), which their cross section data presented in Table 6. Each region has a thickness of 1 mfp. The calculations carried out with our S_*N*_ method for one energy group, 3500 mesh points and N=300 Gauss-Legendre points, and the convergence criterion was $10^{-8}$. Table 7 shows the effective multiplication factor of the multi-region slab geometry using GFM, PARTISN (PARallel TIme Dependent SN transport code (Alcouffe et al., 2000)) and DANT methods. The GFM (using Green's function and angular quadrature's) calculates integrals with error of $10^{-5}$ (Kornreich and Parsons, 2004). The DANT computations use an angular quadrature order of S_96_, and a convergence criterion of $10^{-8}$ (Alcouffe et al., 1995). Fig. 9 shows the comparison of scalar flux variation results calculated by our code and those of GFM method. These results indicate that the approach applied in the present work is able to solve multi-region criticality problems efficiently.

**Table 5:** Structure of the different configuration for multi-region problem.

	Region 1	Region 2	Region 3	Region 4	Region 5	Region 6	Region 7
configuration 1	Reflector	fuel	Reflector	fuel	Reflector	fuel	Reflector
configuration 2	Reflector	fuel	Reflector	fuel	Reflector	Absorber	Reflector
configuration 3	Reflector	fuel	Reflector	fuel	Absorber	fuel	Reflector

**Table 6:** Cross-section data for the one-group multi-region slab geometry.

Material	*ν*Σ_*f*_ (cm^−1^)	Σ_*s*_ (cm^−1^)	Σ_*t*_ (cm^−1^)
Fuel	0.178	0.334	0.415
Reflector	0.0	0.334	0.371
Absorber	0.0	0.037	0.371

**Table 7:** Eigenvalues for different configuration.

Case	S${}_{\text{N}}$	GFM	DANT	PARTISN
configuration 1	1.17361	1.17361	1.17361	1.17361
configuration 2	1.02265	1.02265	1.02265	1.02265
configuration 3	0.94268	0.94268	–	0.94268

According to our results, a good agreement has been obtained between MCNP6 calculations and S${}_{\text{N}}$ method. Indeed the maximum discrepancy doesn't exceed $10^{-8}$. In one-D geometry, we can observe that the angular flux decreases when the neutrons exceed the median plane of the plate. This behavior is to be expected as a consequence of a vacuum boundary conditions which are usually imposed by Mark formalism (Lewis and Miller, 1984), requiring that all ψ(x,μ) with inbound director cosine at a boundary point vanish. The angular flux concept constitutes a very useful reference to test the performance of numerical methods by direct comparison between differential quantities. Comparisons of effective multiplication factors (Table 4, Table 7), scalar flux at different angles (Figs. 5, 6, 7 and 8) which obtained by both S${}_{\text{N}}$ and MCNP6 codes and the scalar flux which calculated by GFM and S${}_{\text{N}}$ methods presented in Fig. 9 confirm the correctness of the implementation of the S${}_{\text{N}}$ method algorithm and the accuracy of our deterministic calculations.

## 4 Conclusions

In this paper, we present quality-of-reference results for angular flux by using our developed one-dimensional neutron transport code based on the S${}_{\text{N}}$ approximation. This approach is based on a symmetrical distribution of the particles circulating in all directions considering the vacuum at the boundary, that's why the Figs. 5, 6, 7 and 8 represent only the right-going ordinates scalar flux at different angles, where the angular flux is zero at the left side. In addition, a new detailed calculation scheme with a matrix formalism of the S${}_{\text{N}}$ method was provided in the purpose of facilitating its implementation in our code written in FORTRAN90. Validation our developed code was performed by comparing S${}_{\text{N}}$ approximation results, of four benchmark suites in single region slab, and within a multi-region slab geometry, to those calculated by MCNP6 Monte Carlo code and GFM method, respectively. Therefore, in our calculation, the effects of spatial discretization on the results of this code were virtually eliminated and the agreement with MCNP6 and GFM results is perfect (less than $10^{-5}$) for high orders of the angular coordinates. The good agreement obtained confirms the accuracy of our S${}_{\text{N}}$ code and the quality of our calculations.

## Declarations

### Author contribution statement

M. Lahdour: Conceived and designed the experiments; Performed the experiments; Analyzed and interpreted the data; Contributed reagents, materials, analysis tools or data; Wrote the paper.

T. El Bardouni: Conceived and designed the experiments; Performed the experiments; Analyzed and interpreted the data; Wrote the paper.

M. Mohammed: Analyzed and interpreted the data; Contributed reagents, materials, analysis tools or data; Wrote the paper.

S. El Ouahdani: Contributed reagents, materials, analysis tools or data; Wrote the paper.

### Funding statement

This research did not receive any specific grant from funding agencies in the public, commercial, or not-for-profit sectors.

### Competing interest statement

The authors declare no conflict of interest.

### Additional information

No additional information is available for this paper.
